# Supplementary material for: Characterizing Microbiomes via Sequencing of Marker Loci: Techniques To Improve Throughput, Account for Cross-Contamination, and Reduce Cost
Source: mSystems. 2021 Jul 13;6(4):e00294-21. doi: 10.1128/mSystems.00294-21 (PMC8409480; doi:10.1128/mSystems.00294-21)
Supplement: TEXT S1 [file msystems.00294-21-t0001.pdf]

Appendix 1: 16s & ITS Illumina Library Preparation SOP

Genome Technologies Laboratory
University of Wyoming

April 16, 2021

1 Introduction

This standard operating procedure (SOP) was inspired by the approach developed by the Genome Sequencing and Analysis Facility at the University of Texas (`https://wikis.utexas.edu/display/GSAF/Home+Page`).

This SOP is for amplification of the 16s (515–806 primer pair) and ITS (ITS1f–ITS2 primer pair) loci from environmental samples, but it could be modified to amplify many other marker loci, simply by changing primer sequences and possibly making minor modifications to the temperatures used during PCR. Libraries made with this protocol are to be sequenced on Illumina platforms, including the iSeq, MiSeq, HiSeq, and NovaSeq.

The SOP makes use of 96 unique "coligos" for tracking cross-contamination among wells (see main text). We also use a synthetic DNA internal standard (ISD) inspired by (Tourlousse et al. 2017). We suggest that future iterations of this SOP be amended to include an internal standard comprising several unique sequences (see Harrison et al. 2020).

Users should be aware that coligo reads will have poly-G tails that arise from a lack of signal from the sequencing machine. These tails are readily trimmed off during bioinformatics or used as a way to identify and remove coligos from the dataset. We also note that coligos, due to their short length, do not always merge well. Therefore, when performing analyses to determine the extent of cross-contamination we recommend using forward reads only. Alternatively, coligos can be synthesized such that they are longer and will merge more readily, however this will increase the cost of coligo synthesis.

2 Materials

- Coligos for sample tracking. Coligo structure: 5'- forward primer - 13 nt coliqo sequence - rear primer - 3'. See `https://github.com/JHarrisonEcoEvo/Genome_Technologies_lab_of_Univ_Wyoming` for suggested coligo sequences.

  - E.g., 16S Coligo: 5'-GTGCCAGCAGCCGCGGTAA AACAACAA-CAACC ATTAGATACCCTAGTAGTCC-3'
  - E.g., ITS Coligo: 5' - CTTGGTCATTTAGAGGAAGTAAT AACAA-CAACAACC CGTAGCTACTTCTTGCGTCG-3'
- Synthetically designed DNA as an internal standard (ISD) or "spike-in". This is a shortened version of the sequence presented in (Tourlousse et al. 2017). Structure: 5'- forward primer - synthetic sequence - rear primer - 3'
  - E.g., ITS ISD: CTTGGTCATTTAGAGGAAGTAATGCCACAGAT-ACGTACCGCTCATAACGCGAACCGAAG CGCAGTAGAAGTACTC-CGTATCCTACCTCGGTCGTGGTTTAGGCTATCGACATCTTG-CATGGGCTTCCCTAGTGAAC TCTTGGGATGTGCATCGAT-GAAGAACGCAGC
  - E.g., 16S ISD: GTGCCAGCAGCCGCGGTAAGCCACAGATACG-TACCGCTCATAACGCGAACCGAAGCGCA GTAGAAGTACTC-CGTATCCTACCTCGGTCGTGGTTTAGGCTATCGACATCTTG-CATGGGCTTCCCTAGTGAACTCTT GGGATGTATTAGATACC-CTAGTAGTCC
- 5X KAPA HiFi HotStart PCR Buffer
- 10M dNTPs
- Kapa HiFi HotStart DNA Polymerase
- HPLC grade H_2O
- Axygen AxyPrep Mag PCR Clean-up Kit
- Template DNA. Ideally, 30 µL of 10 ng or higher concentration.
- Barcoded locus-specific primers. Primers are described at `https://github.com/JHarrisonEcoEvo/Genome_Technologies_lab_of_Univ_Wyoming` and should be arrayed in 96-well plates, for most applications.

  For the one-step procedure, primer design is as follows: 5'- Illumina flow cell adapter (either forward or reverse) - variable length barcode - primer (either forward or reverse) - 3'.

  For the two-step procedure, primer design is the same except only a part of the Illumina flow cell adapter is included.
  - 16S Primer (used for both one and two-step procedures)
    * 515F (5'-GTGYCAGCMGCCGCGGTA-3') (Parada et al. 2016)
    * 806R (5'-GGACTACNVGGGTWTCTAAT-3') (Apprill et al. 2015)
  - ITS Primer (used for both one and two-step procedures)
    * ITS1F (5'-CTTGGTCATTTAGAGGAAGTAA-3') (Gardes and Bruns 1993)

    * ITS2 (3'-CGTAGCTACTTCTTGCGTCG-5') (White et al. 1990)

- Flow cell primers for use with the two-step procedure. These primers extend the barcoded primers during the second round of PCR so that the full Illumina flow cell adapter is added to each amplicon.
  - 2nd round flow cell primer sequences:
    * AATGATACGGCGACCACCGAGATCTACACTCGTCGGCAGCGTC
    * CAAGCAGAAGACGGCATACGAGATGTCTCGTGGGCTCGG

3 Protocol

We present two protocols here, a one-step procedure that adds Illumina flow cell adapters and barcodes during template amplification and a two-step procedure that allows for the use of shorter oligos but requires two rounds of PCR (for visual description see main text). The two-step procedure adds a portion of the Illumina flow cell adaptor and the barcode during initial priming and amplification of the template. A second round of PCR adds the remaining portion of the Illumina adaptor.

The two-step procedure is commonly used because it requires short, inexpensive oligos. However, it takes longer to complete and, for best results, requires an additional PCR clean-up step, which adds significant cost. We have shifted to a one-step approach which has a much lower per-reaction cost and requires less time. The one-step approach requires longer oligos than the two-step technique and these longer oligos incur a large initial cost of a few thousand USD (at the time of writing). However, for laboratories processing many samples, this large initial cost will rapidly be defrayed due to time and consumable savings (e.g., in magnetic bead clean-up kits, pipette tips, and so on).

3.1 A note on cleanliness

Every effort should be made to reduce contamination and keep a clean work space. The work bench should be cleaned with a solution that degrades DNA between projects. Nucleic acid extraction and sample preparation (e.g., the weighing of soil) should be done in a different room from PCR, if at all possible, to minimize the chance of contamination. If a different room is not available, then at least perform extractions and PCR on separate benches and clean all equipment thoroughly when shifting from performing extractions to performing PCR. Pipettes and other equipment should be cleaned daily, or more often, as needed. Ideally, PCR should be performed under a hood. Gloves should be worn during sensitive steps, such as when reagents and MID-plates are opened. Gloves must be clean, one cannot touch anything that has not been cleaned and expect the gloves to perform their function (e.g., cabinet handles, writing tools, keyboards, if they haven't been cleaned then one's gloves will be contaminated). Do not pipette directly from stock solutions.

Follow all other laboratory rules and keep careful notes. Labeling of samples is the most important step of this entire process. Without proper labels all is lost!

3.2 One-step procedure

1. Aliquot 30 µL of full concentration extracted DNA into a new 96 well plate (unskirted, standard well depth). Save this plate in the event the library needs prepared again. Label the plate. We currently advise performing PCR in duplicate.

2. Add 6 µL of control oligo pool to environmental DNA aliquot. The control pool includes 16S and ITS coligos (0.01 pg μL^{-1} of each) and 0.03 pg μL^{-1} each of the 16S and ITS internal standards. If desired, quantify DNA concentration for each sample and normalize. Currently, we are normalizing to 10 ng μL^{-1}. Save aliquot of DNA in case the library needs to be prepared again. Label the plate.

3. Add 11 µL of Master Mix to each well of a new plate (see Table 1 for Master Mix ingredients). Label the plate.

Table 1: Ingredients for Master Mix for one-step procedure. Include 10–15% extra to account for adhesion of liquid to plastics.

µL/rxn	Reagent
3	5X KAPA HiFi HotStart PCR Buffer
0.45	10M dNTPs
0.3	Kapa HiFi HotStart DNA Polymerase
7.25	HPLC grade H_2O
11	Total volume

4. Add 2 µL of the appropriate 0.25 µM primer plate to the plate with the Master Mix.

5. Add 2 µL of template DNA (normalized) to each well of the plate with the Master Mix and primers. After template addition, we recommend sealing the plate well using strip caps to prevent evaporation (if unfamiliar with PCR then experiment with the plastic consumables, including plates and caps, that work best for the equipment available.).

6. Apply the following PCR recipe:

Table 2: PCR conditions for one-step procedure

Temp.	Cycles	Time
95°	1X	3:00 min
98°	36X	30 sec
62°	36X	30 sec
72°	36X	30 sec
72°	1X	5:00 min
4°	1X	hold

7. Pool PCR duplicates together into one of the two plates.

8. Purify samples using the following modified AxyPrep MagBead PCR Clean-Up protocol.

8.1 Equilibrate beads to room temperature.

8.2 Add 24 µL of MagBeads to each well; Mix via pipetting, use 10 aspiration/dispensation cycles.

8.3 Incubate at room temperature for 5 minutes.

8.4 Secure plate on magnet plate; incubate at room temperature for 5 more minutes (ensure wells are clear).

8.5 Remove 65 µL from each well while avoiding the bead pellet. The pellet will stick to the side of the well due to the action of the magnet. The pellet includes the DNA.

8.6 Add 100 µL 80% ethanol to each well and incubate 30 seconds then remove 100 µL from each well.

8.7 Duplicate the last step; i.e., add 100 µL 80% ethanol to each well, incubate 30 seconds, remove 100 µL from each well.

8.8 Aspirate from each well again to assure maximum ethanol removal. Ethanol can interfere with PCR. Visually check the bottoms of the wells by looking up through the bottom of the plate. Sometimes ethanol can remain in a few wells and it is worth attempting its removal.

8.9 Allow plate to air dry for 7 minutes. This allows remaining ethanol to evaporate. If ethanol has not evaporated then try to pipette it out and dry longer until all ethanol has been removed.

8.10 Remove sample plate from magnet plate.

8.11 Add 40 µL TE and pipette mix 10+ times. Incubate 2 minutes at room temperature.

8.12 Place sample plate back on magnet for 5 minutes or until all wells are cleared.

8.13 Remove liquid from the well and place in a new, labeled plate. This is the cleaned DNA. If sequencing is to occur within days or weeks then DNA can be stored in a refrigerator (ensure caps are well sealed). For longer storage, keep DNA in a non-cycling freezer.

3.3 Two-step procedure

1. Aliquot 30 µL of full concentration extracted DNA into a new 96 well plate (unskirted, standard well depth). Save this plate in the event the library needs prepared again. Label the plate. We currently advise performing PCR in duplicate.

2. Add 6 µL of control oligo pool to environmental DNA aliquot. The control pool includes 16S and ITS coligos (0.01 pg μL^{-1} of each) and 0.03 pg μL^{-1} each of the 16S and ITS internal standards. If desired, quantify DNA concentration for each sample and normalize. Currently, we are normalizing to 10 ng μL^{-1}. Save aliquot of DNA in case the library needs to be prepared again. Label the plate.

3. Add 7 µL of Master Mix #1 to each well of a new plate (see Table 3 for Master Mix ingredients). Label the plate.

4. Add 6 µL of the appropriate 0.25 µM barcode plate.

5. Add 2 µL of template DNA (normalized) to each well.

6. Apply the PCR recipe shown in Table 4.

Table 3: Ingredients for Master Mix #1. Include 10–15% extra of reagents to account for wastage due to adhesion of liquid to plastics.

µL/rxn	Reagent
3	5X KAPA HiFi HotStart PCR Buffer
0.45	10M dNTPs
0.3	Kapa HiFi HotStart DNA Polymerase
3.25	HPLC grade H_2O
7	Total volume

Table 4: PCR conditions #1. These conditions are for amplification of the target locus and addition of barcodes.

Temp.	Cycles	Time
95°	1X	3:00 min
98°	15X	30 sec
62°	15X	30 sec
72°	15X	30 sec
72°	1X	5:00 min
4°	1X	hold

7. Pool PCR duplicates together into one of the two plates.

8. Purify samples using the following modified version of the AxyPrep MagBead PCR Clean-Up protocol. Caveat: it may be possible to skip this PCR-clean procedure to reduce costs. Instead, a single clean-up could be done at the end of the protocol. Alternatively, the single-step PCR we describe should be considered, as it only requires a single clean-up.

8.1 Equilibrate beads to room temperature.

8.2 Add 24 µL of MagBeads to each well; Mix via pipetting, use 10 aspiration/dispensation cycles.

8.3 Incubate at room temperature for 5 minutes.

8.4 Secure plate on magnet plate; incubate at room temperature for 5 minutes (ensure wells are clear).

8.5 Remove 65 µL from each well, while avoiding the pellet. The pellet will stick to the side of the well due to the action of the magnet. The pellet contains the DNA.

8.6 Add 100 µL fresh 80% ethanol to each well and incubate 30 seconds. Remove 100 µL from each well.

8.7 Duplicate the last step. I.e., add 100 µL fresh 80% ethanol to each well, incubate 30 seconds, remove 100 µL from each well.

8.8 Aspirate from each well again to assure maximum ethanol removal. Ethanol can interfere with PCR. Visually check the bottoms of the wells by looking up through the bottom of the plate. Sometimes ethanol can remain in a few wells and it is worth trying to remove it.

8.9 Allow plate to air dry for 7 minutes. This allows the remaining ethanol to evaporate. If the ethanol has not evaporated then try to remove it and extend

the drying time until all ethanol has been removed.

8.10 Remove the sample plate from the magnet plate.

8.11 Add 30 µL H_2O; pipette mix 10+ times. Incubate 2 minutes at room temperature.

8.12 Place the sample plate back on magnet for 5 minutes.

8.13 Remove the liquid from the well and place in a new, labeled plate. This is the cleaned DNA.

8.14 Prepare flow cell Master Mix (Table 5).

Table 5: Ingredients for flow cell Master Mix (Mix # 2). Include 10–15% extra of reagents to account for wastage due to adhesion.

µL/rxn	Reagent
3	5X KAPA HiFi HotStart PCR Buffer
0.45	10M dNTPs
0.3	Kapa HiFi HotStart DNA Polymerase
0.5	10 µM of each of the forward and reverse flow cell primers
0.75	HPLC grade H_2O
7	Total volume

9. Add 5 µL flow cell Master Mix to a new plate.

10. Transfer 10 µL cleaned DNA to the new plate that has the flow cell Master Mix.

11. Apply PCR recipe #2 (Table 6) to this plate (which contains the flow cell Master Mix and the cleaned template from PCR #1).

Table 6: PCR conditions #2. These conditions are for addition of Illumina flow cell adapters.

Temp.	Cycles	Time
95°	1X	3:00 min
98°	19X	30 sec
55°	19X	30 sec
72°	19X	30 sec
72°	1X	5:00 min
4°	1X	hold

12. Clean amplicons using the modified MagBead protocol listed below. Note that this differs slightly from the clean up step listed above in that the final step uses TE instead of water.

12.1 Equilibrate beads to room temperature.

12.2 Add 15 µL of H_2O to each well.

12.3 Add 24 µL of MagBeads (0.8 x 30 µL) to each well; Mix via pipetting, use 10 aspiration/dispensation cycles.

12.4 Incubate at room temperature for 5 minutes.

12.5 Secure plate on magnet; incubate at room temperature for 5 minutes (until wells are clear).

12.6 Remove 65 µL from each well, while avoiding the bead pellet, which contains the DNA.

12.7 Add 100 µL fresh 80% ethanol to each well and incubate 30 seconds then remove 100 µL from each well.

12.8 Duplicate the last step. I.e., add 100 µL fresh 80% ethanol to each well, incubate 30 seconds, remove 100 µL from each well.

12.9 Aspirate from each well again to assure maximum ethanol removal. Ethanol can interfere with PCR.

12.10 Allow plate to air dry for 7 minutes. This allows remaining ethanol to evaporate.

12.11 Remove sample plate from magnet plate.

12.12 Add 30 µL TE (Tris ethylenediaminetetraacetic acid); pipette mix 10+ times. Incubate 2 minutes at room temperature.

12.13 Place sample plate back on magnet for 5 minutes.

12.14 Transfer 30 µL to a clean PCR plate.

13. Spotcheck PCR reaction success using an Agilent Bioanalyzer 2100 or equivalent.

14. Optional: Normalize all samples to 9 ng μL^{-1} prior to sequencing. This step is not needed if high sequencing depth is likely since sufficient data should be

obtained from all samples.

15. Optional: Check molar concentration of the pooled library via qPCR.

16. If sequencing is to be performed in days or weeks then store library in refrigerator. If sequencing will not occur for awhile then store the library in a non-cycling freezer.

References

Apprill, A. et al. (2015). "Minor revision to V4 region SSU rRNA 806R gene primer greatly increases detection of SAR11 bacterioplankton". *Aquatic Microbial Ecology* 75.2, pp. 129–137.

Gardes, M. and T. D. Bruns (1993). "ITS primers with enhanced specificity for basidiomycetes - application to the identification of mycorrhizae and rusts". *Molecular Ecology* 2.2, pp. 113–118.

Harrison, J. G. et al. (2020). "The quest for absolute abundance: the use of internal standards for DNA-based community ecology". *Molecular Ecology Resources* n/a (n/a).

Parada, A. E., D. M. Needham, and J. A. Fuhrman (2016). "Every base matters: assessing small subunit rRNA primers for marine microbiomes with mock communities, time series and global field samples". *Environmental Microbiology* 18.5, pp. 1403–1414.

Tourlousse, D. M. et al. (2017). "Synthetic spike-in standards for high-throughput 16S rRNA gene amplicon sequencing". *Nucleic Acids Research* 45.4, e23–e23.

White, T. J. et al. (1990). *Amplification and direct sequencing of fungal ribosomal RNA genes for phylogenetics. In M. A. Innis, D. H. Glefand, J. J. Sninsky, and T. J. White [eds.], PCR protocols: A guide to methods and applications.* London, UK: Academic Press. 315-322.
