# Supplementary material for: Characterizing Microbiomes via Sequencing of Marker Loci: Techniques To Improve Throughput, Account for Cross-Contamination, and Reduce Cost
Source: mSystems. 2021 Jul 13;6(4):e00294-21. doi: 10.1128/mSystems.00294-21 (PMC8409480; doi:10.1128/mSystems.00294-21)
Supplement: TABLE S1 [file msystems.00294-21-st001.pdf]

Table S1: The proportion of reads belonging to non-target coligos in each well of three 96-well plates (denoted as replicates in upper left of each table). Each well was seeded with only one coligo. If an unexpected coligo sequence was observed within a well then this was due to cross-contamination. Cross-contamination was very minor in all cases. Also, see Figs. 3, S2–S4

. We observed that H12 was slightly contaminated in each replicate, which was suggestive of minor cross-contamination during coligo stock preparation.

Rep. 1	1	2	3	4	5	6	7	8	9	10	11	12
A	1.000	1.000	1.000	1.000	1.000	1.000	1.000	1.000	1.000	1.000	1.000	1.000
B	0.999	1.000	1.000	1.000	1.000	1.000	1.000	1.000	1.000	1.000	1.000	0.999
C	1.000	1.000	1.000	1.000	1.000	1.000	1.000	1.000	1.000	1.000	1.000	1.000
D	1.000	1.000	1.000	1.000	1.000	1.000	1.000	1.000	1.000	1.000	1.000	1.000
E	1.000	1.000	1.000	1.000	1.000	1.000	1.000	1.000	1.000	0.999	1.000	1.000
F	1.000	1.000	1.000	1.000	1.000	1.000	1.000	1.000	1.000	1.000	1.000	1.000
G	1.000	1.000	1.000	1.000	1.000	1.000	1.000	1.000	1.000	1.000	1.000	1.000
H	1.000	1.000	1.000	1.000	1.000	1.000	1.000	1.000	1.000	1.000	1.000	0.999
Rep. 2	1	2	3	4	5	6	7	8	9	10	11	12
A	1.000	1.000	1.000	1.000	1.000	1.000	1.000	1.000	1.000	1.000	1.000	0.999
B	1.000	1.000	1.000	1.000	1.000	1.000	1.000	1.000	1.000	1.000	1.000	1.000
C	1.000	1.000	1.000	1.000	1.000	1.000	1.000	1.000	1.000	1.000	1.000	1.000
D	1.000	1.000	1.000	1.000	0.999	1.000	1.000	1.000	1.000	1.000	1.000	1.000
E	1.000	1.000	1.000	1.000	1.000	1.000	1.000	1.000	1.000	1.000	1.000	1.000
F	1.000	1.000	1.000	1.000	1.000	1.000	1.000	1.000	1.000	1.000	1.000	1.000
G	1.000	1.000	1.000	1.000	1.000	1.000	1.000	1.000	1.000	1.000	1.000	1.000
H	1.000	1.000	1.000	1.000	1.000	1.000	1.000	1.000	1.000	1.000	1.000	0.999
Rep. 3	1	2	3	4	5	6	7	8	9	10	11	12
A	1.000	1.000	1.000	1.000	1.000	1.000	1.000	1.000	1.000	1.000	1.000	1.000
B	1.000	1.000	1.000	1.000	1.000	1.000	1.000	1.000	1.000	1.000	1.000	1.000
C	1.000	1.000	1.000	1.000	1.000	1.000	1.000	1.000	1.000	0.999	1.000	1.000
D	1.000	1.000	1.000	1.000	1.000	1.000	1.000	1.000	1.000	1.000	1.000	1.000
E	1.000	1.000	1.000	1.000	1.000	1.000	1.000	1.000	1.000	1.000	1.000	1.000
F	1.000	1.000	1.000	1.000	1.000	1.000	1.000	1.000	1.000	1.000	1.000	1.000
G	1.000	1.000	1.000	1.000	1.000	1.000	1.000	1.000	1.000	1.000	1.000	1.000
H	1.000	1.000	1.000	1.000	1.000	1.000	1.000	1.000	1.000	1.000	1.000	0.998
