# Supplementary figures and images for: Characterizing Microbiomes via Sequencing of Marker Loci: Techniques To Improve Throughput, Account for Cross-Contamination, and Reduce Cost
Source: mSystems. 2021 Jul 13;6(4):e00294-21. doi: 10.1128/mSystems.00294-21 (PMC8409480; doi:10.1128/mSystems.00294-21)

a)

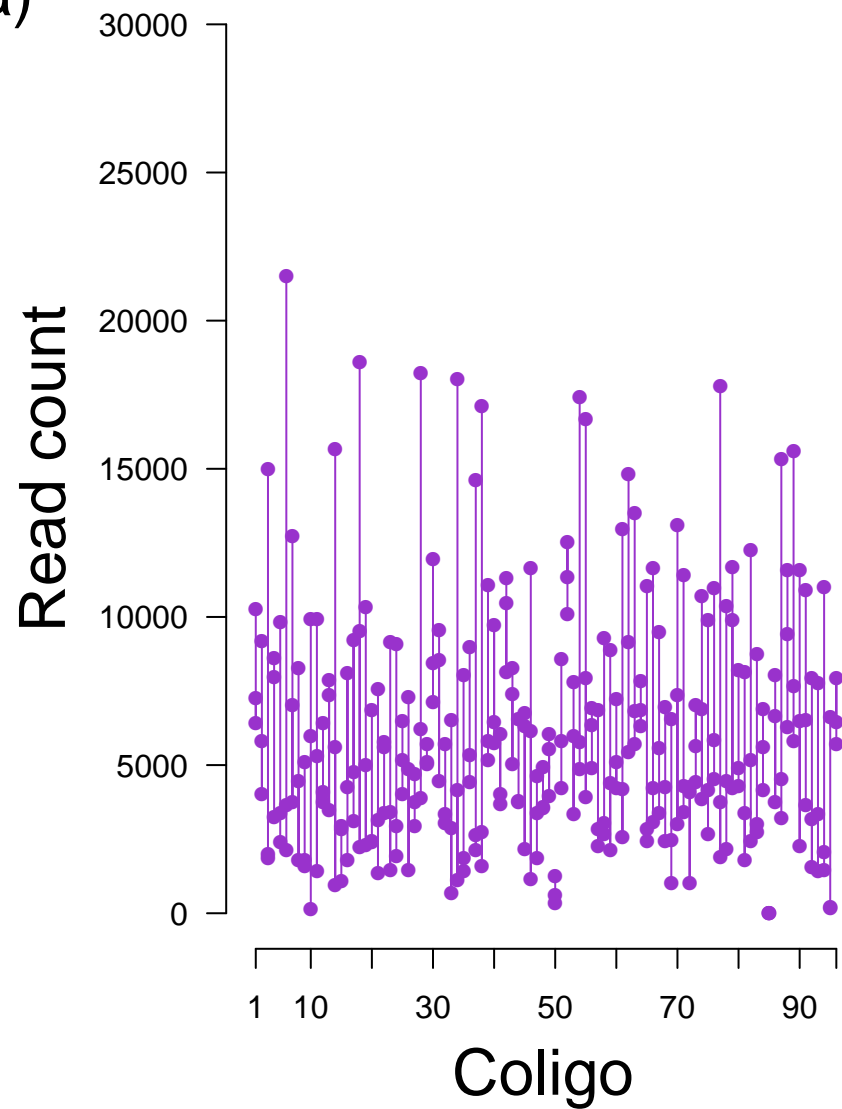

b)

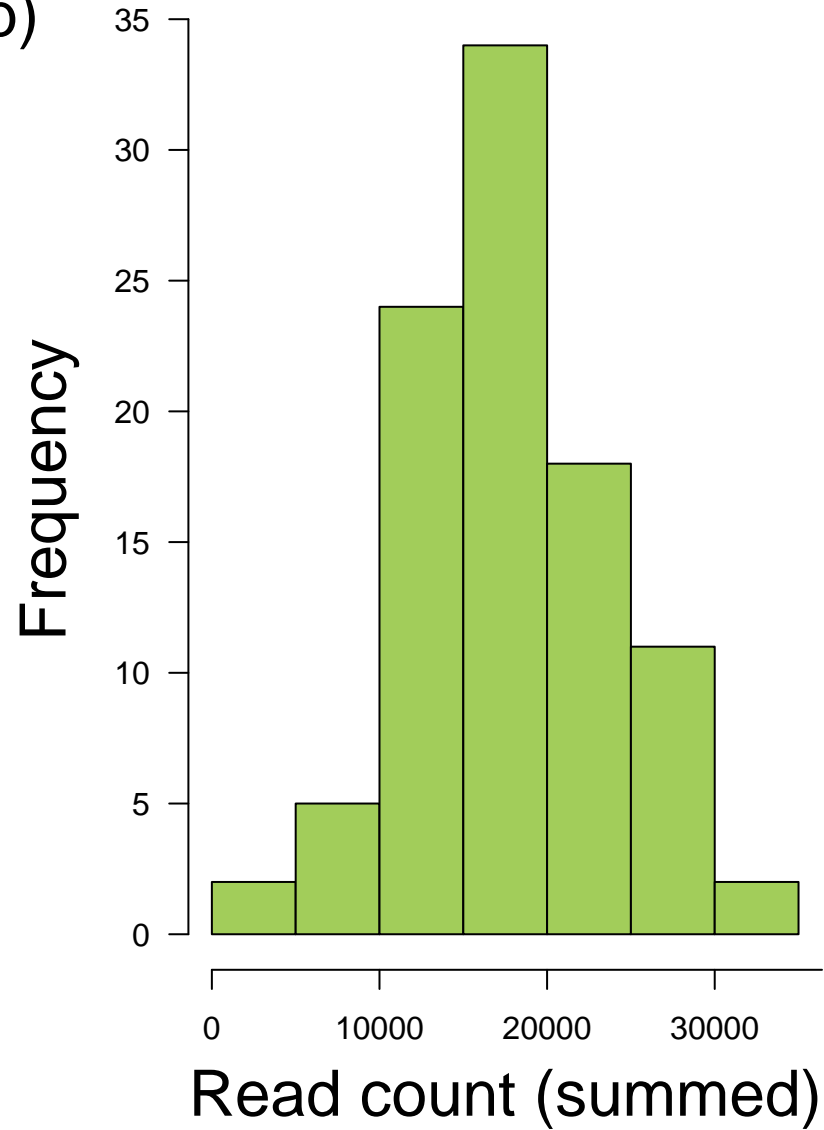

Supplement: FIG S2 [file msystems.00294-21-sf002.pdf]

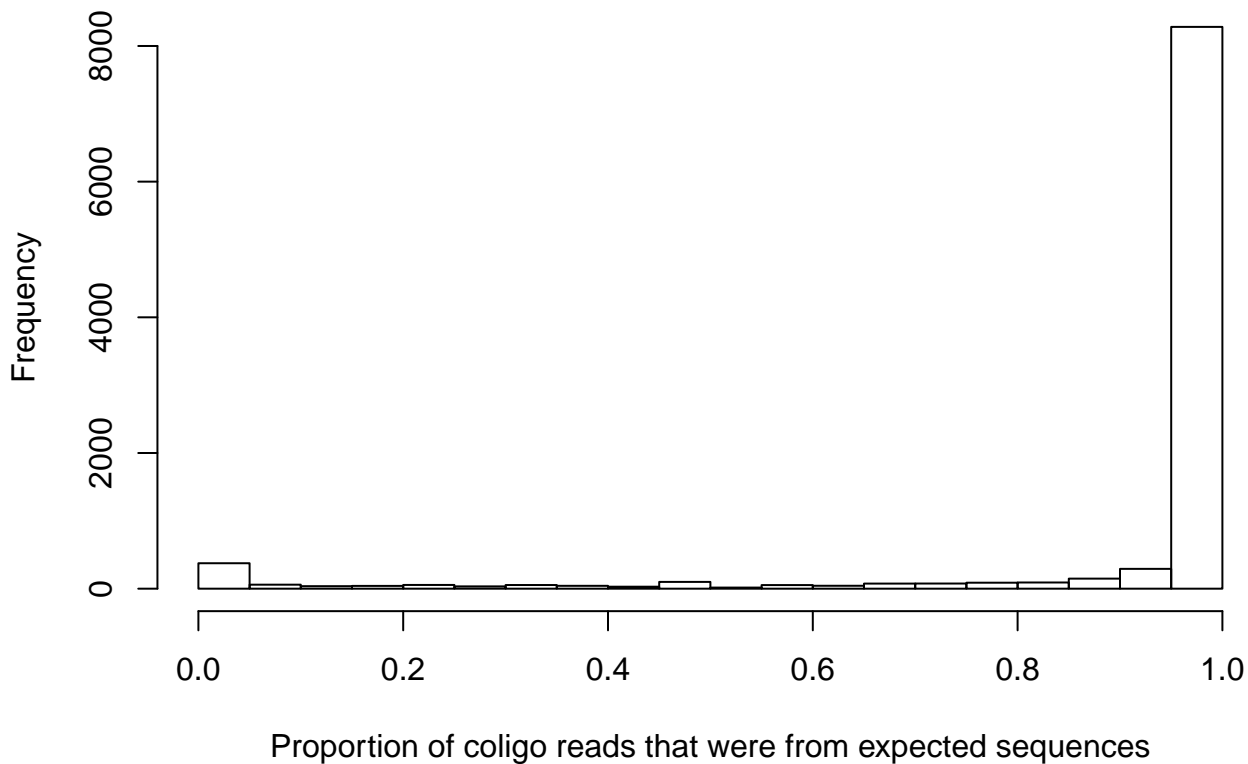

Supplement: FIG S3 [file msystems.00294-21-sf003.pdf]

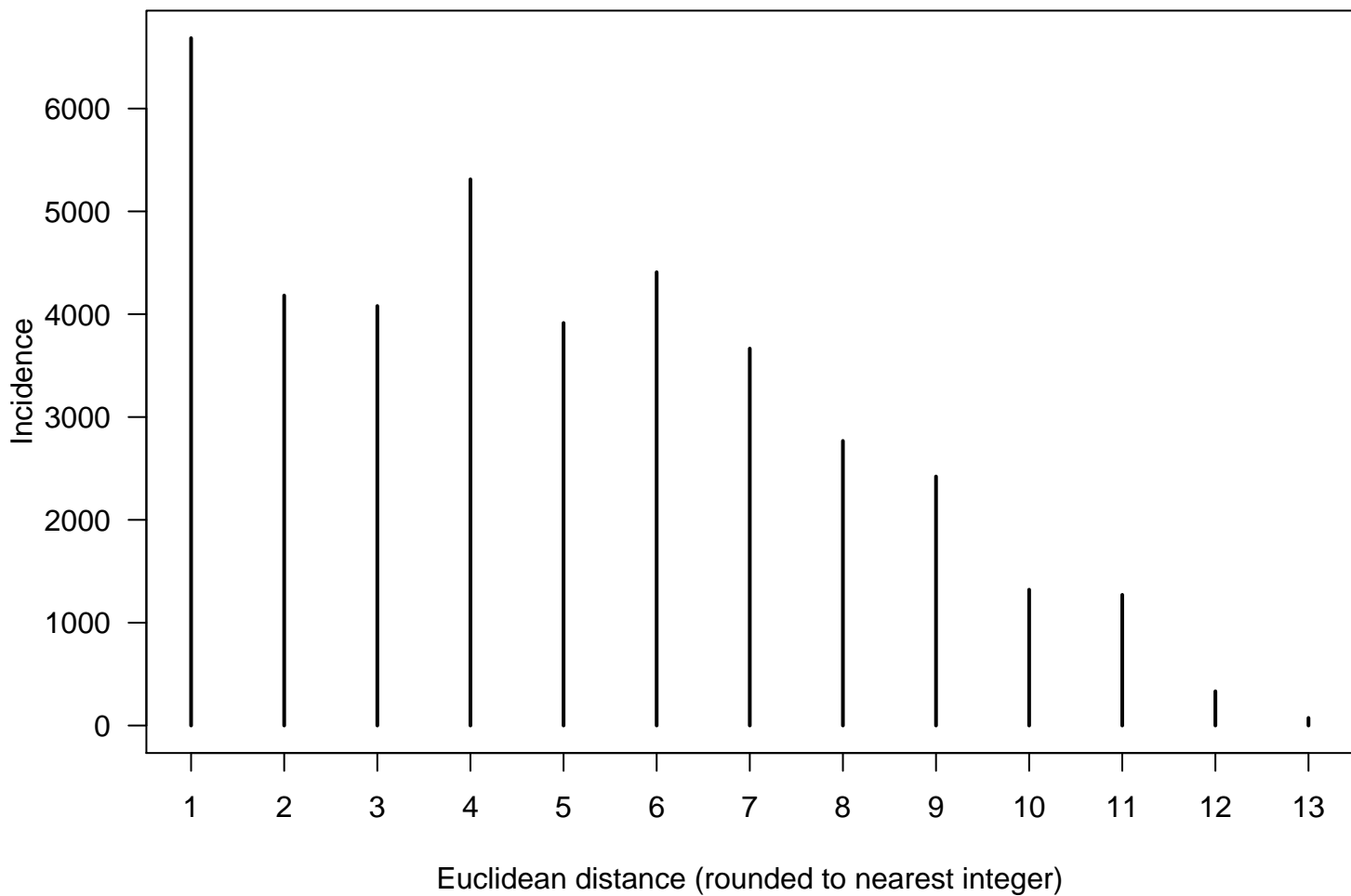

Supplement: FIG S4 [file msystems.00294-21-sf004.pdf]

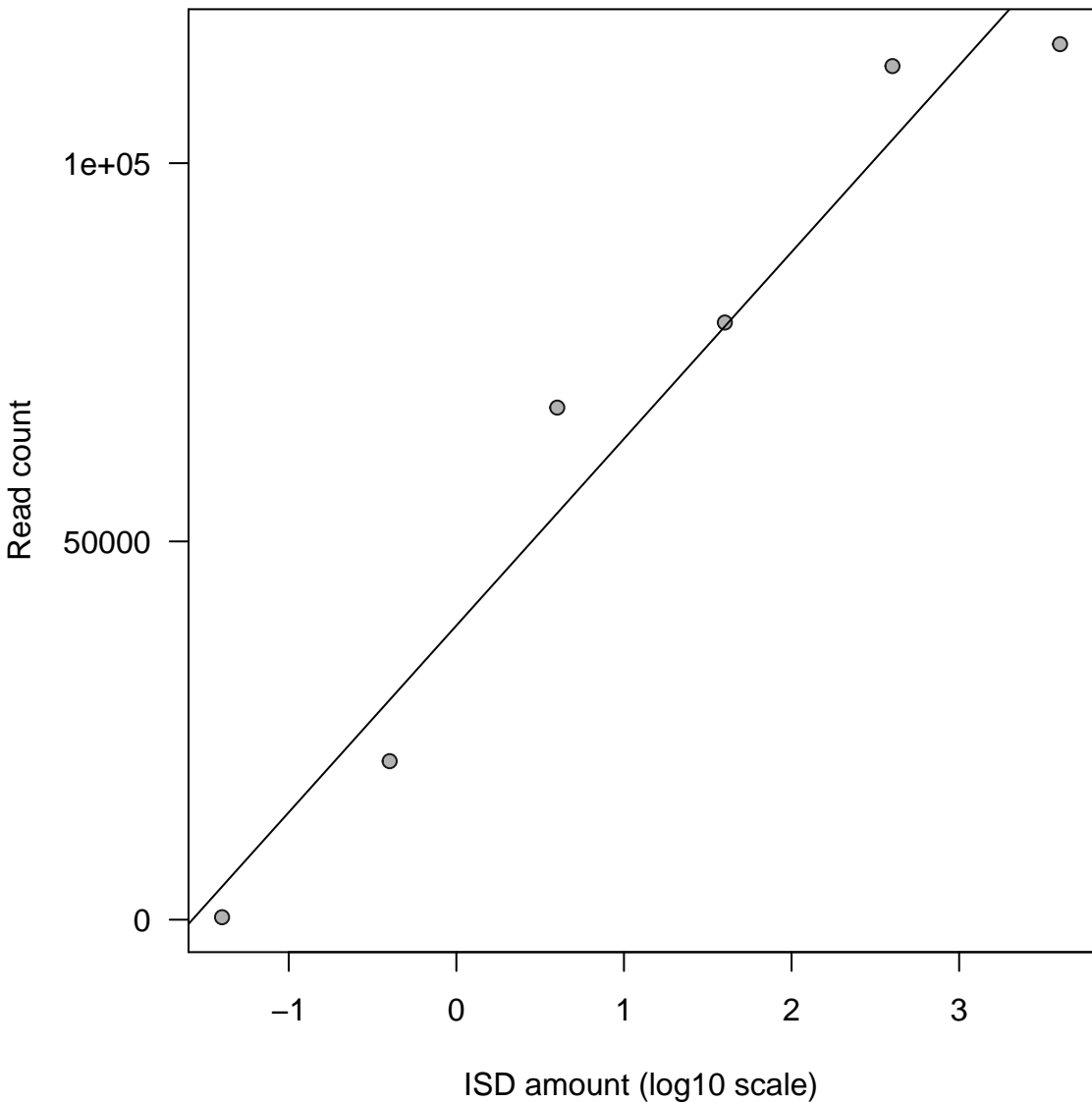

Supplement: FIG S1 [file msystems.00294-21-sf001.pdf]
