# Supplementary material for: Characterizing Microbiomes via Sequencing of Marker Loci: Techniques To Improve Throughput, Account for Cross-Contamination, and Reduce Cost
Source: mSystems. 2021 Jul 13;6(4):e00294-21. doi: 10.1128/mSystems.00294-21 (PMC8409480; doi:10.1128/mSystems.00294-21)
Supplement: TEXT S2 [file msystems.00294-21-t0002.pdf]

Library preparation details for sequencing experiments

Libraries for both the iSeq and the NovaSeq were prepared following the protocols described in Appendix 1. iSeq sequencing was performed following the manufacturer's directions.

Bioinformatics

Sequences were demultiplexed using a custom Perl script that corrected for up to one substitution error in MIDs using Levenshtein distances. For the iSeq library containing only coligo, internal standard (ISD), and mock community sequences, we performed analyses on forward reads only, because read lengths were too short to allow reads to merge, and because analysis of rear reads were not required to meet the experimental goal of testing coligo performance.

To make a table linking sequence variants to replicates, we used the USEARCH v10.0.240 [4] suite of software. Primer regions were removed from forward reads (via removing a fixed number of bases) and then filtered to remove any reads with more than a single expected error using USEARCH. We note that NovaSeq machines provide a binned quality score that is different from earlier Illumina machines. Thus dada2 [1] and other denoisers, including USEARCH, are challenged by the new quality data. No clear consensus has yet emerged for how to best deal with binned quality scores. In our view, the dramatic output provided by the NovaSeq makes it the sequencing machine of choice, despite the challenge posed by binned quality scores.

Unique, filtered reads were clustered into exact sequence variants (ESVs) using UNOISE3 as implemented by USEARCH [5]. ESVs that were low complexity, matched PhiX, or were less than 64 nt long were removed. An ESV table was created by matching original unfiltered reads to each ESV using the "otutab" command of USEARCH. Taxonomic hypotheses were assigned via SINTAX [3] through comparison to the Greengenes database [accessed January 2020; 2] as implemented via VSEARCH [8]. The "usearch_global" function was used to match ESV sequences to the ISD sequence.

The same general bioinformatics approach was used to process the example NovaSeq library that was created using our two-step PCR procedure and the library created to test the efficacy of our one-step procedure except that paired reads were merged and analyses were primarily conducted on merged reads.

Because our coligos are very short sequences, they do not merge well and were filtered out early in the bioinformatic pipeline described above. Coligos do not merge well due to a phenomenon known as staggering, which occurs when the 3' end of forward reads extends well past the 5' end of the overlapped reverse read, or vice versa. Therefore, we processed coligo sequence data slightly differently. We removed primers using cutadapt v2.10 [6] and then matched the following 13 bases to our coligo sequences using the "search_exact" function of vsearch. The resulting table showed the number of times a particular coligo was counted within a replicate. We also note that some alignment algorithms perform poorly with very short sequences, so we currently advocate searching for coligos using exact matching. As oligo synthesis costs are reduced, it may be beneficial to synthesize longer coligos to streamline bioinformatics.

Statistical analysis and plotting was conducted in the R computing environment [7].

References

[1] B. J. Callahan et al. “DADA2: High-resolution sample inference from Illumina amplicon data”. *Nature Methods* 13.7 (2016), pp. 581–583.

[2] T. Z. DeSantis et al. “Greengenes, a chimera-checked 16S rRNA gene database and workbench compatible with ARB”. *Applied and Environmental Microbiology* 72.7 (2006), pp. 5069–5072.

[3] R. Edgar. “SINTAX: a simple non-Bayesian taxonomy classifier for 16S and ITS sequences”. *bioRxiv* (2016), p. 074161.

[4] R. C. Edgar. “Search and clustering orders of magnitude faster than BLAST”. *Bioinformatics* 26.19 (2010), pp. 2460–2461.

[5] R. C. Edgar. “UNOISE2: improved error-correction for Illumina 16S and ITS amplicon sequencing”. *bioRxiv* (2016), p. 081257.

[6] M. Martin. “Cutadapt removes adapter sequences from high-throughput sequencing reads”. *EMBnet.journal* 17.1 (2011), pp. 10–12.

[7] R Core Team. *R: A language and environment for statistical computing.* Vienna, Austria, 2020.

[8] T. Rognes et al. “VSEARCH: a versatile open source tool for metagenomics”. *PeerJ* 4 (2016).
